# Skeletal Muscle Fibre Type Determines Mitochondrial and Metabolic Responses to Hypoxia and Pulmonary Inflammation in Young Adult Rats

**DOI:** 10.1002/jcsm.70382

**Published:** 2026-09-23

**Authors:** Angelos Gavrielatos, Cécile Cottet‐Rousselle, Noé Brocker, Cindy Tellier, Amel Achouri, Alexandre Prola, Hervé Dubouchaud, Clovis Chabert

**Affiliations:** ^1^ Laboratory of Fundamental and Applied Bioenergetics (LBFA) Université Grenoble Alpes Grenoble France

**Keywords:** hypoxia, mitochondrial function, muscle fibre type, permeability transition pore, pulmonary inflammation, ROS

## Abstract

**Background:**

Chronic obstructive pulmonary disease (COPD) patients often experience skeletal muscle dysfunction that may result from a complex combination of mitochondrial dysfunction, metabolic reprogramming and fibre type transitions. Among other factors, pulmonary inflammation and hypoxia contribute to the COPD‐associated muscle defects. Nevertheless, the precise molecular mechanisms and their effects across muscles with distinct metabolic profiles remain elusive. This study investigated the independent and combined effects of chronic pulmonary inflammation and chronic hypoxia on mitochondrial function, metabolic enzyme activity and fibre‐type composition in oxidative (soleus) and glycolytic (plantaris) muscles.

**Methods:**

Adult male Wistar rats were subjected to 4 weeks of chronic hypoxia (FiO_2_ = 10%) and/or chronic pulmonary inflammation (induced by 1 mg/kg biweekly intratracheal instillations of lipopolysaccharides). Mitochondria from both soleus and plantaris were isolated to measure oxygen consumption rates, reactive oxygen species (ROS) emission, calcium retention capacity (CRC), respiratory complex activities and fatty acid oxidation capacity. Cross‐sections from both muscles were analysed for fibre typology and fibre cross‐sectional area (fCSA) as well as succinate dehydrogenase (SDH) and glycerol‐3‐phosphate dehydrogenase (GPDH) activities.

**Results:**

Chronic hypoxia led to a decline in adenosine diphosphate‐stimulated complex I (CI)–mediated respiration (−37%; *p* < 0.01), Hydroxyacyl‐Coenzyme A dehydrogenase activity (−27%; *p* < 0.01), weight (−17%; *p* < 0.05) and fCSA of Type IIb fibres (−16%; *p* < 0.05) in plantaris. In contrast, chronic hypoxia increased ROS emission (+74%; *p* < 0.01) without changes in mitochondrial respiration or mass in soleus. No alterations in fibre typology were observed in either muscle following the hypoxia exposure. Chronic pulmonary inflammation caused a reduction in mitochondrial CRC (−29%; *p* < 0.001) and an increase in GPDH activity in Type IIa fibres of soleus (+17%; *p* < 0.001) without any changes in fibre type distribution. Conversely, chronic pulmonary inflammation induced a downregulation of GPDH activity in plantaris types I and IIa fibres (−27% and −32%, respectively; *p* < 0.05), in parallel with an elevation in the SDH/GPDH ratio across all fibre types (+73%; *p* < 0.05) and a rise in the proportion of Type IIx fibres (+12%; *p* < 0.05).

**Conclusions:**

Our results demonstrate fundamental differences in the responses to chronic hypoxia and chronic pulmonary inflammation between soleus and plantaris. Although hypoxia affects predominantly the mitochondrial function and mass of plantaris, pulmonary inflammation drives metabolic reprogramming in both muscles that opposes their intrinsic functional specialisation. Additionally, soleus appears more vulnerable to permeability transition pore opening following pulmonary inflammation. Notably, these alterations occurred independently of fibre type transitions, suggesting a role for qualitative mitochondrial alterations in COPD‐associated muscle dysfunction.

## 1 Introduction

Skeletal muscle dysfunction and atrophy are common features in a range of pathological conditions including chronic obstructive pulmonary disease (COPD). Notably, more than 30% of COPD patients experience skeletal muscle impairments, which serve as critical indicators of deteriorated quality of life, exercise intolerance and increased reliance on healthcare resources [1]. To date, accumulating evidence confirms that limb muscles in COPD may exhibit alterations in mitochondrial function and structure including a reduction in mitochondrial density [2], depressed respiratory capacity [3] and a decline in oxidative enzyme activity [4, 5]. This metabolic reprogramming may further be associated with faster opening of the mitochondrial permeability transition pore (mPTP) with concomitant cytochrome c release [6] and elevated reactive oxygen species (ROS) emission [3], collectively pointing to profound mitochondrial remodelling within the COPD‐affected muscle.

The pathogenesis of structural and functional alterations in skeletal muscle of COPD patients is multifactorial involving oxidative stress, pulmonary inflammation, alveolar hypoxia, malnutrition, physical inactivity and corticosteroid administration [7, 8]. However, their individual contributions to skeletal muscle mitochondrial alterations remain unclear. The pulmonary origin of COPD establishes the lungs as the primary driver of the skeletal muscle mitochondrial pathology likely through chronic airway inflammation, which may propagate to the muscle via spill‐over of inflammatory mediators [9], and alveolar hypoxia, which may lead to muscle tissue hypoxia [10], initiating peripheral muscle wasting before secondary confounders (e.g., physical inactivity) exacerbate it. Chronic hypoxia has been more extensively investigated for its impact on skeletal muscle mitochondria, with the available data suggesting an elevation in ROS production [11], a decrease in mitochondrial respiration [12] and a shift towards glycolytic metabolism [13]. Yet distinguishing these effects from the compounding influence of physical inactivity remains challenging, as many studies may have examined hypoxia concurrently with inactivity. Furthermore, variation in parameters of hypoxic exposure (e.g., continuous vs. intermittent, duration and fraction of inspired O_2_ [FiO_2_]) complicates cross‐study comparisons [14]. In contrast, despite the well‐studied role of inflammation in muscle homeostasis [15], the consequences of chronic pulmonary inflammation on skeletal muscle mitochondria are poorly defined with current evidence largely confined to acute models.

We have previously demonstrated that chronic pulmonary inflammation blunts the overload‐induced hypertrophy in the oxidative rat soleus [16], whereas de Theije et al. [17] have reported that 21 days of hypoxic exposure leads to atrophy, independently of hypophagia, in glycolytic mice extensor digitorum longus but not in soleus. Considering the divergent mitochondrial signatures between oxidative and glycolytic muscles, especially with respect to respiratory properties, ROS metabolism and mPTP regulation by Ca^2+^ [18], mitochondrial responses to inflammation and hypoxia may also present a fibre type‐specific heterogeneity. Nevertheless, neither study assessed mitochondrial function or metabolic parameters under these conditions. Therefore, elucidating the differential susceptibility of glycolytic and oxidative muscles to these stressors remains imperative for uncovering mechanisms underlying compartmentalised muscle dysfunction and atrophy, while guiding the development of phenotype‐specific rehabilitation, personalised antiatrophy interventions and biomarker discovery.

Facing these limitations, the aim of the current study was to unravel the isolated and combined effects of chronic pulmonary inflammation and chronic hypoxia on oxidative soleus and glycolytic‐leaning plantaris muscles in adult male rats with a focus on mitochondrial function and key metabolic enzymes. Given that fibre type transitions are commonly observed in skeletal muscle of COPD patients [19], we also performed a fibre typology analysis in both muscles to explore whether any mitochondrial changes are accompanied by alterations in the myosin heavy chain (MHC) phenotype. Based on prior findings from our laboratory, we hypothesised that the highly oxidative soleus, with its dense capillary network, would exhibit greater sensitivity to pulmonary inflammation due to enhanced perfusion of systemic inflammatory mediators, whereas the glycolytic‐leaning plantaris, with sparser vascularisation, would develop more pronounced pathological alterations under chronic hypoxia.

## 2 Methods

### 2.1 Animals

The study was conducted in accordance with the European Union Directive (2010/63/EU) on laboratory animal care and use and received approval from the local Ethics Committee (Cometh 12) affiliated with the laboratory and authorised by the French Ministry of Higher Education, Research and Innovation (APAFIS #40584‐2023013114463378). Twenty‐eight young adult male Wistar rats aged 20–27 weeks with mean body weight of 530 ± 41 g were evenly allocated according to weight and age across the different experimental groups (mean age at sacrifice: 27 weeks). The animals were housed in individual cages with free access to food (Ref: 3430; SAFE, Augy, France) and water. Body mass and food intake were recorded weekly.

**FIGURE 1:**
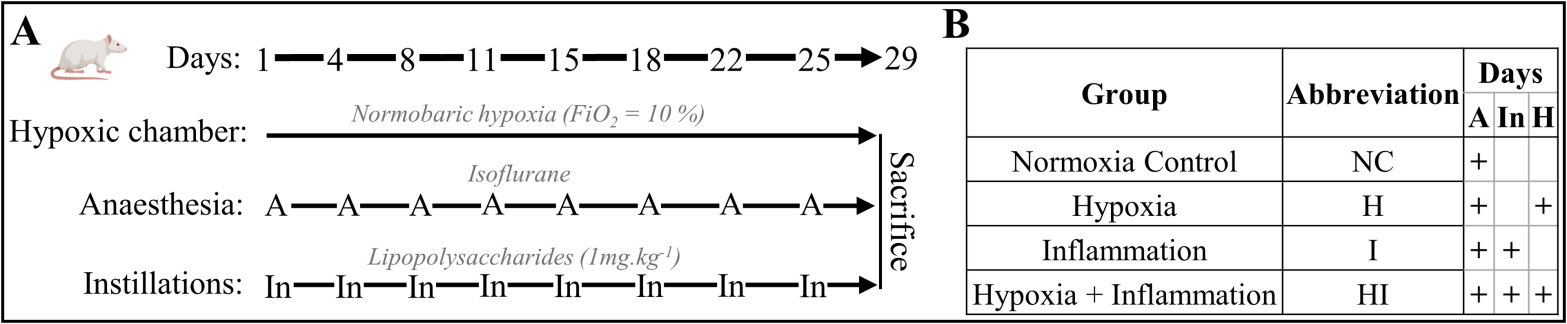
Experimental design of the study. (A) Timeline of the different procedures carried out. (B) Summary table of the procedures performed on animals according to their group. A, anaesthesia; In, intratracheal instillations.

Rats were assigned to one of the following groups for 28 days until sacrifice: (1) normoxia control (NC), (2) chronic hypoxia (H), (3) chronic pulmonary inflammation (I) and (4) H + I (HI). An overview of the experimental design is provided in Figure 1. Chronic pulmonary inflammation was induced by intratracheal instillations of lipopolysaccharides (LPS) at 1 mg/kg of body weight ( *E. coli* , serotype O55:B5; Sigma‐Aldrich Chemical Co., St Louis, Missouri, United States) in NaCl 0.9% twice per week for 4 weeks after isoflurane anaesthesia. The final LPS instillation occurred ≥ 60 h prior to sacrifice to preclude confounding effects by acute‐phase inflammatory responses. Animals from the H and HI groups were exposed for 28 days to normobaric hypoxia in O_2_‐controlled chambers (FiO_2_: 10%). The hypoxic chambers were opened for 10–15 min each day to weigh the animals, the food pellets and to perform LPS instillations to the rats from the HI group. This model combining chronic hypoxia and inflammation has been validated in previous studies from our laboratory [16, 20]. At the end of the study, animals were sacrificed by exsanguination via the abdominal aorta following intraperitoneal injection of ketamine and xylazine (80 mg/kg and 50 mg/kg of body weight, respectively). Soleus and plantaris were bilaterally dissected, with right hindlimb muscles processed for isolation of mitochondria, whereas left hindlimb muscles were cryopreserved in liquid nitrogen‐cooled isopentane and stored at −80°C until cryosectioning.

### 2.2 Mitochondria Isolation

Immediately after dissection, soleus and plantaris were immersed in ice‐cold isolation buffer (150 mM saccharose, 75‐mM KCl, 50‐mM tris base, 1‐mM KH_2_PO_4_, 5‐mM MgCl_2_, 1‐mM EGTA and pH 7.4) and trimmed of connective tissue before transfer to isolation buffer supplemented with 0.2% fat‐free bovine serum albumin (BSA) at 4°C. Muscles were minced with scissors and incubated with subtilisin (0.24 mg/mL) for 1 min before addition of isolation buffer and 0.2% BSA to stop the reaction. The suspension was subsequently homogenised using a motor‐operated glass‐Teflon pestle homogeniser and centrifuged twice at 800 g for 10 min at 4°C to remove cell debris and nuclei. Mitochondria from the resulting supernatant were pelleted by centrifugation at 8000*g* for 10 min at 4°C. The mitochondrial pellet was resuspended in 0.2‐mL respiration buffer (125 mM KCl, 20 mM Tris Base, 1 mM EGTA, pH 7.2) and kept on ice until use. The supernatant containing the cytosolic fraction was also collected and stored at −80°C until spectrophotometric analysis of lactate dehydrogenase activity (LDH). Mitochondrial protein concentration was determined using the bicinchoninic acid assay (BCA; Pierce, Thermo Fisher Scientific, Waltham, MA, United States) with BSA standards (Pierce).

### 2.3 Oxygen Consumption Measurement

To assess the effects of hypoxia and inflammation on the function of the electron transport chain and their individual complexes using specific substrates and inhibitors, all measurements in this study were performed on isolated mitochondria. Mitochondrial oxygen consumption was measured using a Clark‐type O_2_ electrode (Oxygraph+, Hansatech Instruments, Pentney, United Kingdom). Mitochondria (0.2 mg/mL) were incubated in air‐saturated respiration buffer supplemented with 0.15% BSA and 0.1‐M inorganic phosphate (Pi) at 30°C, under constant electromagnetic stirring. Mitochondria were energised using CI (glutamate/malate, GM; 5 mM/2.5 mM) and Complex II (CII) substrates (succinate, S; 5 mM) either alone or in combination (GMS). Oxygen consumption was recorded before (state 2) and after addition of 1‐mM adenosine diphosphate (ADP) (state 3) and following the addition of oligomycin (1 μg/mL) (State 4). When succinate was utilised as the sole substrate, rotenone (4 μM) was added to inhibit CI. The activity of cytochrome c oxidase (Complex IV, CIV) was determined by sequential additions of antimycin A (AA; 2 μΜ), ascorbate (2.5 mM), TMPD/ascorbate (0.5 mM/0.2 mM) and finally the uncoupler 2,4 dinitrophenol (150 μM). Mitochondrial respiration rates were expressed as nmol O/min/mg mitochondrial protein.

### 2.4 Mitochondrial H_2_O_2_ Release

Mitochondrial hydrogen peroxide (H_2_O_2_) release was quantified with a fluorimeter (F2500 Hitachi, Tokyo, Japan) by monitoring the linear increase in fluorescence due to oxidation of Amplex Red to resorufin (excitation, ex, at 560 nm; emission, em, at 584 nm) by H_2_O_2_ in the presence of horseradish peroxidase. Isolated mitochondria (0.2 mg/mL) were incubated at 30°C in mitochondrial respiration buffer, supplemented with 0.15% BSA, 6 U/mL of HRP and 1 μM of Amplex Red under constant electromagnetic stirring. Mitochondrial H_2_O_2_ emission was assessed in basal conditions with GM (5 mM/2.5 mM) or S (5 mM), followed by addition of rotenone (3 μM). AA (3 μM) was also added following rotenone when GM was used as a substrate. Results were expressed as pmol H_2_O_2_/min/mg mitochondrial protein using a known amount of H_2_O_2_ as a standard.

### 2.5 Calcium Retention Capacity

To assess mPTP sensitivity, the mitochondrial calcium retention capacity (CRC) was quantified using a spectrofluorimeter (Horiba PTI QM‐400 Special QuantaMaster, Irvine, California, United States). Mitochondria (0.1 mg/mL) were incubated at 30°C under continuous electromagnetic stirring in 250‐mM saccharose, 10‐mM Pi, 10‐mM Tris‐MOPS, 10‐mM EGTA and pH 7.4, containing 125‐μM calcium green‐5 N (Calcium Green‐5 N, Thermo Fisher Scientific) as the Ca^2+^‐sensitive fluorescent probe. Mitochondria were energised with GM with or without 1‐μM cyclosporin A (CsA), which is an inhibitor of mPTP. Ca^2+^ pulses (1.5 nmol for soleus, 2 nmol for plantaris) were added at 1‐min intervals. CRC was calculated as the total amount of Ca^2+^ (nmol/mg protein) required to trigger opening of the mPTP, indicated by a sharp increase in fluorescence due to Ca^2+^ release from the mitochondrial matrix.

### 2.6 Respiratory Chain Complex I, HAD and LDH Activities

To determine CI activity, mitochondria (10 μg/mL) were incubated at 30°C in 47.5 mM KH_2_PO_4_, pH 7.5, containing 95 μM EDTA, 3.75 mg/mL BSA, 100 μM NADH and 100 μM decylubiquinone. NADH oxidation kinetics were monitored at 340 nm for 4 min with or without the presence of 10‐μM rotenone to calculate total and nonspecific activities, respectively. To assess HAD activity, mitochondria (10 μg/mL) were incubated at 30°C in 47.5 mM KH_2_PO_4_, pH 7.5 supplemented with 200‐μM NADH, 60‐μM EDTA and 40‐mM imidazole. Following initial measurement of nonspecific activity, total HAD activity was quantified by monitoring the decrease in the NADH absorbance at 340 nm after addition of acetoacetyl‐CoA (3‐ketoacyl‐CoA analogue). LDH activity was determined in plantaris cytosolic fractions obtained during mitochondrial isolation. After determination of protein concentration using the BCA assay, samples were incubated at 37°C in 100‐mM TRIS buffer, pH 7.1, 15‐mM NADH. Following initial measurement of nonspecific activity, total LDH activity was quantified by monitoring the decrease in the NADH absorbance at 340 nm after addition of pyruvate. CI, HAD and LDH specific activities were expressed as μmol/min/mg protein.

### 2.7 Histochemistry and Immunofluorescence

Muscle cross‐sections (8 μm) were cut at −20°C (Leica CM3050 S, Wetzlar, Germany) and mounted on SuperFrost Plus slides (Epredia, NH, United States). Slides were air‐dried at room temperature (RT) and stored at −20°C. All slides were stained within 1 month after cryosectioning.

The activity of succinate dehydrogenase (SDH) and glycerol‐3‐phosphate dehydrogenase (GPDH) was determined histochemically. Freshly prepared and prewarmed at 37°C, either SDH (50‐mM sodium succinate, 0.45‐mM 1‐methoxyphenazinum methosulfate, 5‐mM sodium azide and 4.95‐mM nitroblue tetrazolium chloride) or GPDH (50‐mM Racemic G1P, 1‐mM menadione, 5‐mM sodium azide and 4.95‐mM nitroblue tetrazolium chloride) reaction medium, diluted in 0.1 M Tris‐maleate buffer (pH 7.5) with 10% polyvinyl alcohol, was applied to tissue sections. Enzyme reactions proceeded for 10 min at 37°C under light‐protected conditions and were stopped with ice‐cold PBS. The specificity of the staining was validated using competitive inhibitors: 50‐mM dihydroxyacetone phosphate for GPDH and 250‐mM malonate for SDH.

To determine muscle fibre typology and fibre cross‐sectional area (fCSA), slides were subsequently incubated for 1 h at 37°C with primary antibodies targeting MHC isoforms diluted in PBS, 1% goat serum and 2% BSA. Soleus samples were immunolabelled with antibodies against MHC I (BA‐F8 IgG2b, 1:50) and MHC IIa (SC‐71 IgG1, 1:100) isoforms. Plantaris specimens were incubated with antibodies against MHC I (BA‐F8, 1:100), MHC IIa (SC‐71, 1:200) and MHC IIb (BF‐F3 IgM, 1:100), whereas the MHC IIx isoform was identified by exclusion. All the antibodies were obtained from the Developmental Studies Hybridoma Bank (University of Iowa, IA, United States). Species‐ and subclass‐specific fluorescent secondary antibodies, purchased from Invitrogen (Waltham, MA, United States), were then incubated with slides for 1 h at 37°C (Alexa Fluor 488, Reference: A21141, 1:300 for BA‐F8; Alexa Fluor 568, Reference: A21124, 1:300, for SC‐71; Alexa Fluor 647, Reference: A21238, 1:300, for BF‐F3). Following subsequent PBS washes, cellular membranes were stained with CF405S WGA (1:200 CliniSciences, Nanterre, France) for 10 min at RT. Slides were air‐dried, mounted with ProLong Gold Antifade (Invitrogen), and cured overnight at RT under light‐protected conditions.

### 2.8 Image Acquisition and Analysis

Images were acquired within 24 h postmounting using a Leica DMi8 wide‐field epifluorescence microscope (Leica Microsystems, Wetzlar, Germany) equipped with a 20×/0.8 dry objective and a camera (Leica DFC 9000GT). Raw data collection and processing were done using the manufacturer's LAS‐X software. Fluorescence signal was recorded with four filter cubes (CF405S WGA: DAPI cube, λexc = 390 nm, λem = 435 nm; Alexa Fluor 488: 3‐GFP cube, λexc = 475 nm, λem = 519 nm; Alexa Fluor 568: 5‐Rhodamine cube, λexc = 555 nm, λem = 594 nm; Alexa Fluor 647, 7‐Cy5 cube, λexc = 635 nm, λem = 641 nm). SDH and GPDH enzyme activities were imaged using brightfield microscopy on the same Leica DMi8 microscope (illumination intensity: 100%). All morphological analyses and subsequent quantification were conducted using Fiji (v1.54m), an enhanced distribution of ImageJ (National Institute of Health, United States) by two investigators blinded to experimental group coding.

To determine fibre type classification, an assisted automatic detection of the fibres based on WGA membranes staining was performed and the mean signal intensity in each channel was measured. For each channel, a threshold was established, beyond which fibres were classified as positive for a given MHC isoform. Fibre typology was determined by manually counting all fibres within entire muscle cross‐sections. The abundance of each fibre type was calculated as a percentage of the total fibre count. Fibre cross‐sectional areas were determined by encircling WGA‐stained cell membranes and analysing a minimum of 50 fibres per type except for the soleus Type IIa fibres. To determine the mean fCSA of each group, mean fCSA of each sample was averaged. For the SDH and GPDH analyses, the enzymatic activity was calculated by measuring the integral density (sum of pixel values in a defined area) of each fibre divided over the fCSA and expressed in arbitrary units (AU)/min. Group‐level mean activities for SDH and GPDH were derived by averaging sample means.

### 2.9 Statistical Analysis

Normality of the data was examined using the Shapiro–Wilk test and visual inspection of histograms and boxplots. Nonnormal distributions were normalised with log₁₀ or square root transformations. To examine the effect of the two experimental conditions (chronic constant hypoxia and chronic pulmonary inflammation), a two‐way analysis of variance (ANOVA) was performed with each of the experimental conditions (Condition 1: normoxia or hypoxia; Condition 2: with or without pulmonary inflammation) as between‐subjects factors. Post hoc analyses for significant interaction effects employed Bonferroni‐corrected one‐way ANOVAs and independent *t* tests. All fibre typology variables were treated as a family of hypotheses due to their inherent interdependence and were analysed using a two‐way multivariate analysis of variance. Model assumptions were evaluated via Box's M test and visual examination of correlation matrices. Data are presented as individual data points with mean and standard deviation (SD). A threshold of *p* < 0.05 was used to define statistical significance. Outliers were defined as individual values located ≥ or ≤ 2 SD from the group mean and were excluded prior to the statistical analysis. Across the dataset, this resulted in the removal of a small number of values (three observations). Effect sizes were quantified by partial eta square (*η* ^2^_*p*_) values, with a *η* ^2^_*p*_ value ≥ 0.14 indicating a large effect. Statistical analyses were conducted with SPSS (v27, IBM, Chicago, IL, United States) and all figures were generated with GraphPad Prism (v8.0.2 GraphPad Software, San Diego, United States) except for Figure 1 that was designed using BioRender.com.

## 3 Results

### 3.1 Body Weight, Food Intake and Muscle Weight

**FIGURE 2:**
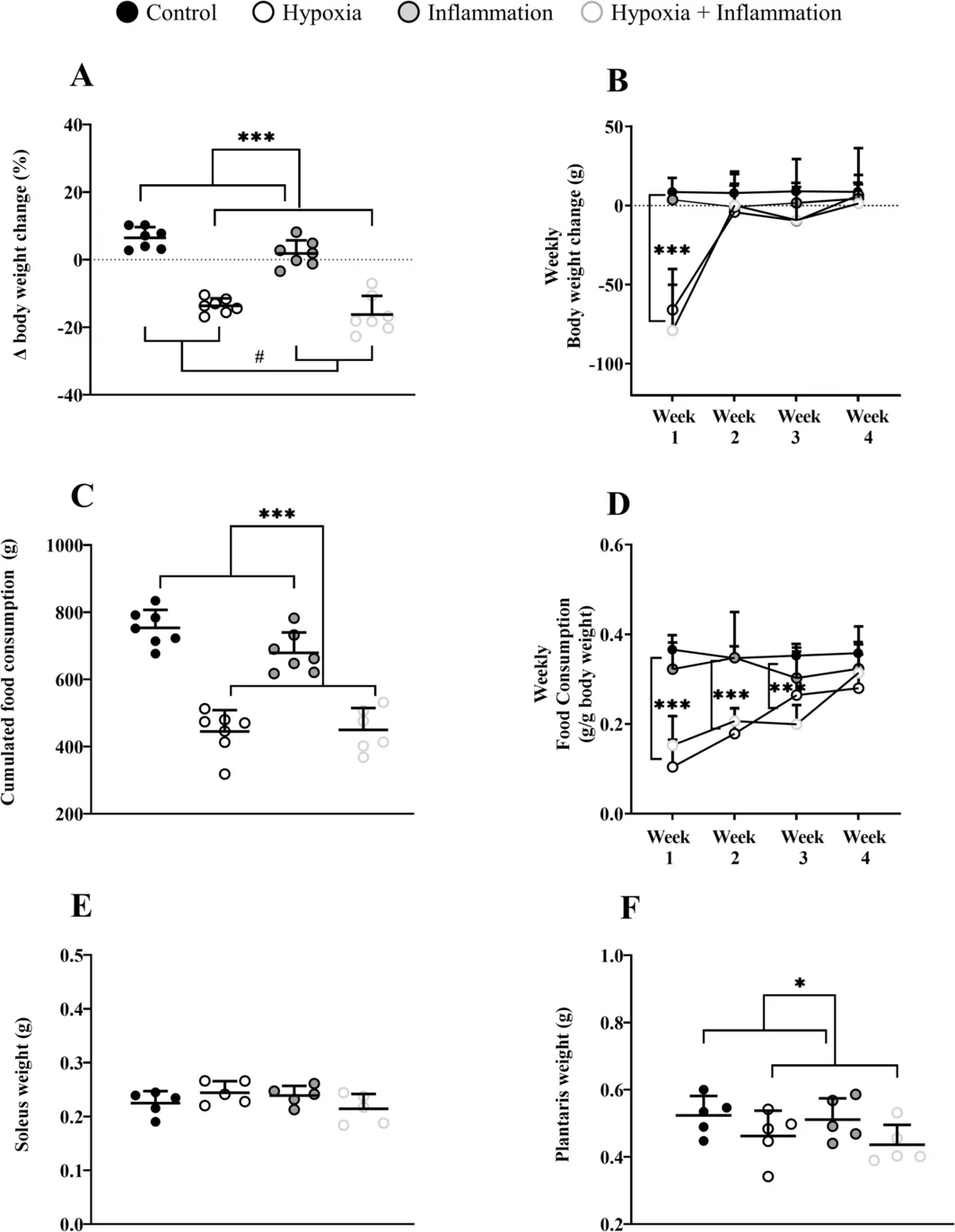
Body mass, food intake and muscle weight under hypoxia and/or inflammation. (A) Change in animal body weight normalised to baseline measurement on Day 1. (B) Weekly changes in body weight throughout the 4‐week exposure. (C) Total cumulated food intake over the 4‐week study period. (D) Weekly changes in the relative food intake of the rats throughout the 4‐week study period. Soleus (E) and plantaris (F) weight at sacrifice. Circles indicate individual data points. Large horizontal lines represent means and error bars indicate SD. *Global effect of hypoxia (NC + I vs. H + HI; *p < 0.05 and ***p < 0.001); ^#^ *p* < 0.05, global effect of pulmonary inflammation (NC + H vs. I + HI).

Body weight evolution during the study revealed a significant decrease associated with chronic hypoxia compared with normoxic conditions (NC + I: +4.14% ± 4% vs. H + HI: −14.9% ± 4%; *p* < 0.001; *η* ^2^_*p*_ = 0.88; Figure 2A) as well as with chronic inflammation compared with non‐LPS–instilled animals (NC + H: −3.6% ± 11% vs. I + HI: −7.2% ± 10%; *p* < 0.05; *η* ^2^_*p*_ = 0.20; Figure 2A). These differences were driven by a marked reduction in body weight in hypoxia‐exposed animals during the first week of exposure (−14.5%; *p* < 0.001; *η* ^2^_*p*_ = 0.82; Figure 2B), whereas no significant weekly changes were observed following exposure to pulmonary inflammation. Body weights were unchanged over the last 3 weeks of exposure in all the groups (Figure 2B). Cumulated food consumption over the study period was lower in animals exposed to hypoxia (NC + I: 716 ± 67.2 g vs. H + HI: 447 ± 61.5 g; *p* < 0.001; *η* ^2^_*p*_ = 0.85; Figure 2C). When adjusted to body mass, weekly food consumption was lower in hypoxia groups compared to normoxia groups during the first 3 weeks of exposure but not during the final week (Week 1: −92%, *p* < 0.001, *η* ^2^_*p*_ = 0.82; Week 2: −58%, *p* < 0.001, *η* ^2^_*p*_ = 0.64; Week 3: −33%, *p* < 0.001, *η* ^2^_*p*_ = 0.39; Week 4: −14%, *p* = 0.132, *η* ^2^_*p*_ = 0.096; Figure 2D). Hypoxia exposure induced a decline in plantaris muscle weight (NC + I: 0.52 ± 0.06 g vs. H + HI: 0.45 ± 0.07 g; *p* < 0.05; *η* ^2^_*p*_ = 0.26; Figure 2F) but not in soleus muscle, when compared to normoxia conditions.

### 3.2 Mitochondrial Respiration

**FIGURE 3:**
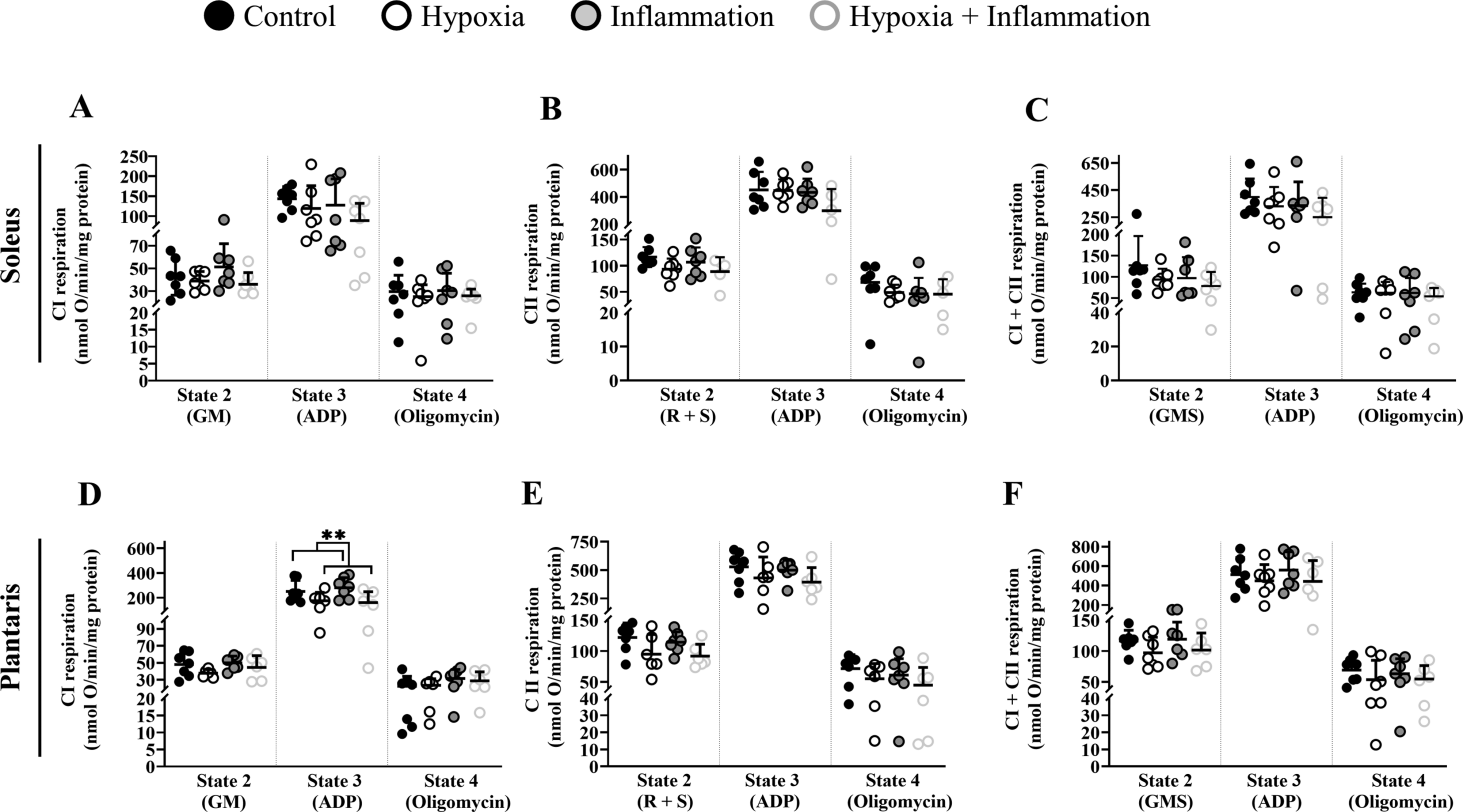
Oxygen consumption in isolated mitochondria from soleus and plantaris muscle after exposure to hypoxia and/or inflammation. Soleus (A–C) and plantaris (D–F) isolated mitochondrial respiration measured by oxygraphy with GM (A, D), succinate + rotenone (B, E) and GMS (C, F) substrates (Basal State 2 condition), after ADP addition (ADP‐stimulated State 3 condition) or after addition of oligomycin (nonphosphorylating State 4 condition). Circles indicate individual data points. Large horizontal lines represent means and error bars indicate SD. Broken *y* axes were used to improve visualisation of values across different respiration states. **p < 0.01, global effect of hypoxia (NC + I vs. H + HI).

Neither condition changed the respiration under basal (substrate alone), State 3 (ADP‐stimulated) or State 4 (nonphosphorylating) conditions in soleus mitochondria, regardless of the substrates fuelling the electron transport chain (Figure 3A–C). Similarly, the respiratory control ratio (RCR: State 3/State 4) was not affected by the exposure to pulmonary inflammation and/or hypoxia in soleus under any condition (Figure S1A). Soleus CIV‐mediated respiration appeared also unaffected (Figure S2A) by inflammation and/or hypoxia. In contrast, chronic hypoxia led to a decline in State 3 respiration with Complex I substrates in mitochondria from plantaris muscle (−45%, *p* < 0.01; *η* ^2^_*p*_ = 0.29; Figure 3D). However, neither hypoxia nor inflammation altered State 2 or 4 CI‐driven respiration (Figure 3D) or RCR (Figure S1B) in mitochondria from plantaris. Furthermore, no changes in plantaris mitochondrial respiration or RCR were observed for any other conditions (Figures 3E–F, S1B, and S2B).

### 3.3 Mitochondrial H_2_O_2_ Release

**FIGURE 4:**
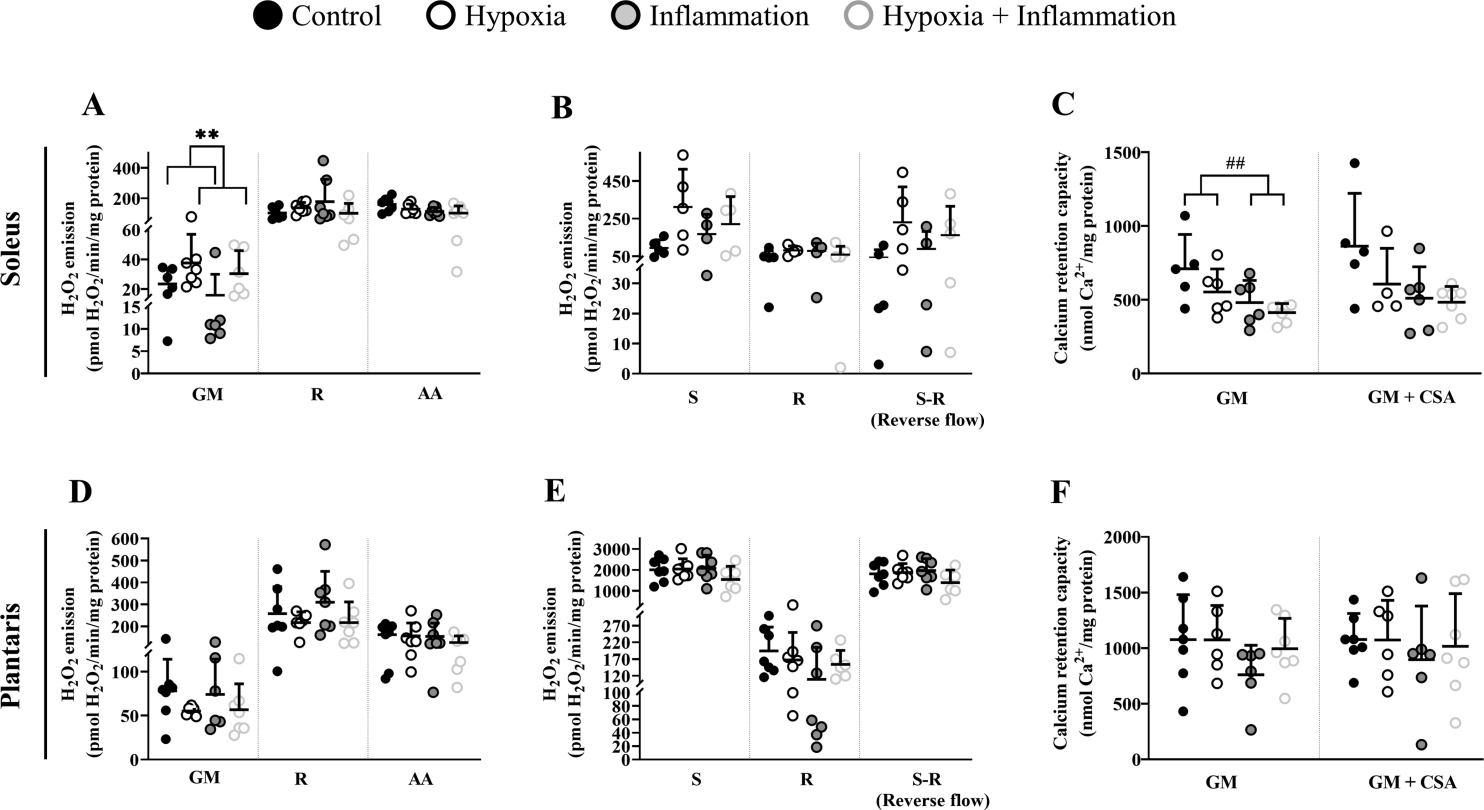
Effect of hypoxia and/or inflammation exposures on isolated mitochondrial H_2_O_2_ emission and calcium retention capacity. Soleus (A, B) and plantaris (D, E) isolated mitochondrial H_2_O_2_ release with substrate alone and after rotenone and AA inhibition. (C, F) Soleus (C) and plantaris (F) isolated mitochondrial CRC was measured with GM or with GM + CsA. Circles indicate individual data points. Large horizontal lines represent means and error bars indicate SD. Broken *y* axes were used in Panels A, B, D and E to improve visualisation of values across experimental conditions. **p < 0.01, global effect of hypoxia (NC + I vs. H + HI) ^##^ *p* < 0.01, global effect of pulmonary inflammation (NC + H vs. I + HI).

Basal mitochondrial H_2_O_2_ release, as an indicator of mitochondrial ROS production, was significantly higher in soleus isolated mitochondria after exposure to chronic hypoxia with GM as substrate (+54.8%, *p* < 0.01, *η* ^2^_*p*_ = 0.39; Figure 4A). Consistently, hypoxia exposure was associated with a higher ratio of H_2_O_2_ emission over basal respiration using GM (+57.6%, *p* < 0.001; *η* ^2^_*p*_ = 0.43; Figure S3A). However, this effect in soleus muscle was abolished when rotenone was used to block CI or when AA was added (Figure 4A). Notably, neither hypoxia nor pulmonary inflammation altered H_2_O_2_ emission in soleus isolated mitochondria, whether measured during combined forward and reverse electron transfer pathways using S or isolated reverse electron flow revealed by CI inhibition with rotenone (Figure 4B). These findings were consistent with the lack of changes in ROS release with succinate as substrate when normalised to CII‐driven basal respiration rate (Figure S3A). In plantaris, hypoxia and inflammation exposures did not have any effect on isolated mitochondria H_2_O_2_ release regardless the combination of substrates and inhibitors (Figures 4D–F and S3B).

### 3.4 Calcium Retention Capacity

Chronic pulmonary inflammation led to a decline in soleus mitochondrial CRC when GM was used to fuel the electron transport chain (NC + H: 624 ± 202 nmol Ca^2+^/mg protein vs. I + HI: 445 ± 112 nmol Ca^2+^/mg protein; *p* < 0.001; *η* ^2^_*p*_ = 0.3; Figure 4C). However, inhibition of the mitochondrial permeability transition pore with CsA reversed this effect of inflammation exposure (Figure 4C). No change in plantaris CRC was detected whether mitochondria were incubated with GM alone or GM combined with CsA (Figure 4F).

### 3.5 CI, HAD and LDH Enzymatic Activities in Isolated Mitochondria

**FIGURE 5:**
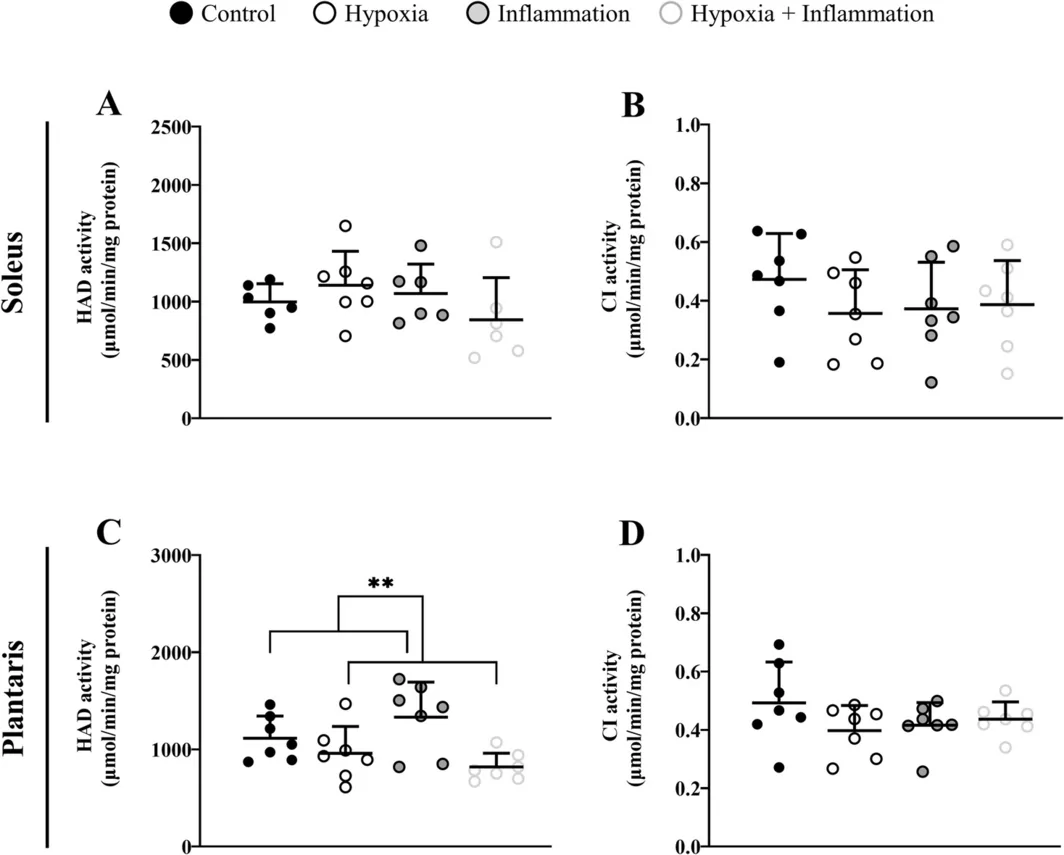
Effect of hypoxia and/or inflammation exposure on isolated HAD activity and CI activity. HAD activity was measured in isolated mitochondria of soleus (A) and plantaris (C). CI activity was measured in isolated mitochondria of soleus (B) and plantaris (D). Circles indicate individual data points. Large horizontal lines represent means and error bars indicate SD. **p < 0.01, global effect of hypoxia (NC + I vs. H + HI).

There was no effect of pulmonary inflammation or hypoxia on HAD activity of soleus mitochondria (Figure 5A). However, exposure to chronic hypoxia led a significant decrease in HAD activity of plantaris mitochondria (−31.5%; *p* < 0.01; *η* ^2^_*p*_ = 0.32; Figure 5C). Neither environmental constraint induced alterations in isolated CI activity in soleus or plantaris muscles (Figure 5B,D). Finally, there was no effect of hypoxia and/or inflammation on plantaris LDH activity (Figure S4).

### 3.6 Fibre Typology and fCSA

**FIGURE 6:**
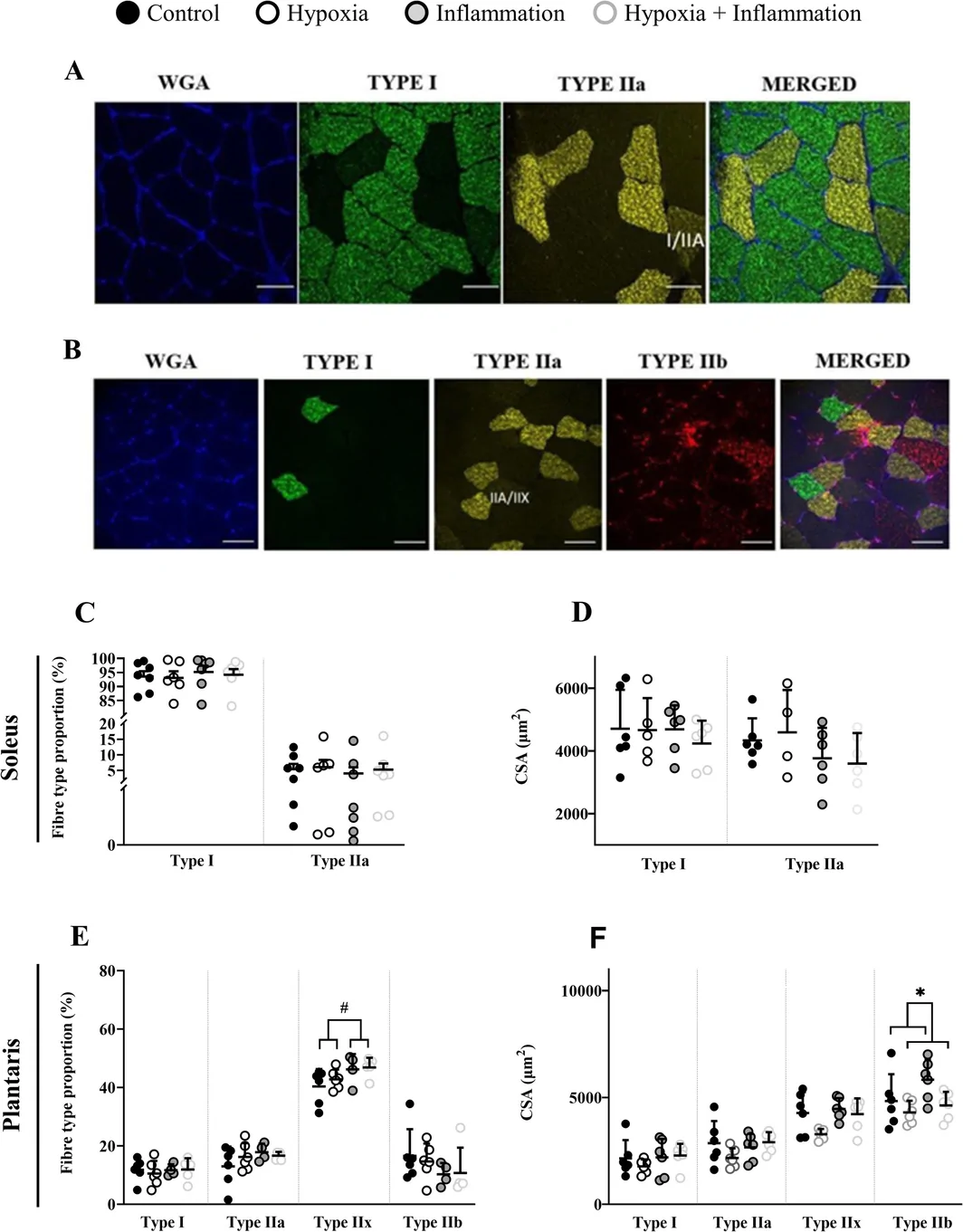
Effects of hypoxia and/or inflammation exposure on fibre typology and cross‐sectional area. (A, B) Representative images of MHC and WGA. Images show WGA, MHC I, MHC IIA and merged (WGA + MHC I + MHC IIa) staining for soleus (A) and WGA, MHC I, MHC IIa, MHC IIb and merged (WGA + MHC I + MHC IIa + MHC IIb) staining for plantaris (B). Scale bar, 50 μm. Percentage of Types I and IIa fibres in soleus (C) and Types I, IIa, IIx and IIb fibres in plantaris (E). CSA was assessed in Types I and IIa fibres of soleus (D) and Types I, IIa, IIx and IIb fibres of plantaris (F). Circles indicate individual data points. Large horizontal lines represent means and error bars indicate SD. Broken *y* axes were used in Panel C to improve visualisation of values across different measurement ranges. ^#^ *p* < 0.05, global effect of pulmonary inflammation (NC + H vs. I + HI). *p < 0.01, global effect of hypoxia (NC + I vs. H + HI).

There were no alterations in the fibre type composition of soleus in response to either intervention (Figure 6C). In contrast, chronic pulmonary inflammation led to a significant elevation in the proportion of Type IIx fibres in plantaris (NC + H: 41.6% ± 4.8% vs. I + HI: 46.6% ± 4%, *p* < 0.05, *η* ^2^_*p*_ = 0.26, Figure 6E). Furthermore, there were no changes in the hybrid fibre population in soleus (Type I/IIa) or in plantaris (Types I/IIa, IIa/IIx and IIx/IIb) following exposure to either stressor (Figure S5). Neither condition affected the Type I or IIa fCSA in soleus muscle (Figure 6D). However, in plantaris, hypoxia led to a decrease in the fCSA of Type IIb fibres but not in Type I, IIa or IIx (Type IIb: NC + I: 5332 ± 1182 μm^2^ vs. H + HI: 4464 ± 591; *p* < 0.05, *η* ^2^_*p*_ = 0.22, Figure 6F).

### 3.7 SDH and GPDH Activities

**FIGURE 7:**
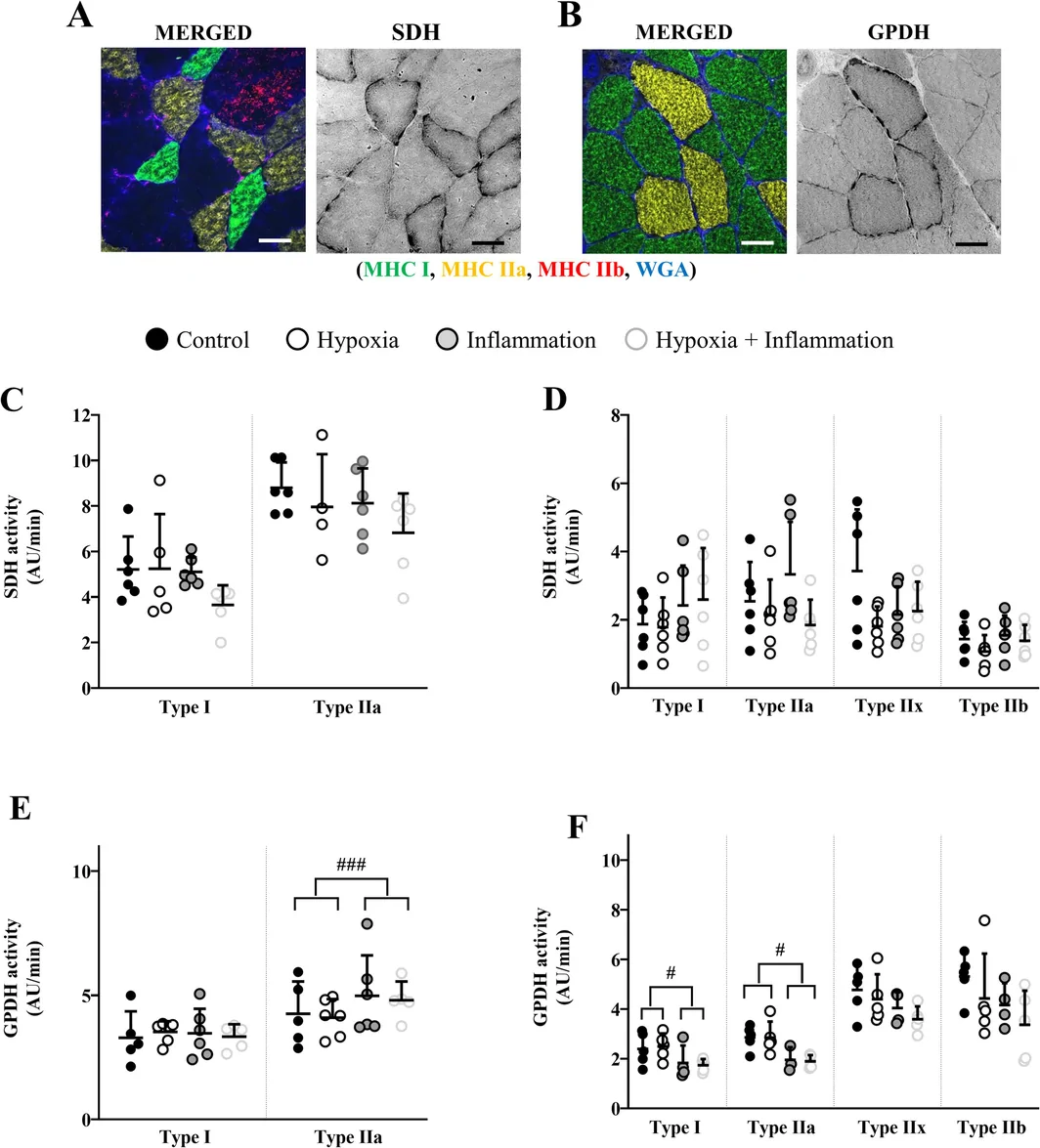
Effects of hypoxia and/or inflammation exposure on SDH and GPDH activity of soleus and plantaris. (A, B) Representative images show merged (WGA + MHC I + MHC IIa + MHC IIb) and SDH staining for plantaris (A) and merged (WGA + MHC I + MHC IIa) and GPDH staining for soleus (B). Scale bar, 50 μm. (C, D) SDH activity in Types I and IIa fibres of soleus (C) and Types I, IIa, IIx and IIb fibres of plantaris (D). (E, F) GPDH activity in Types I and IIa fibres of soleus (E) and Types I, IIa, IIx and IIb fibres of plantaris (F). Circles indicate individual data points. Large horizontal lines represent means and error bars indicate SD. ^#^ *p* < 0.05, global effect of pulmonary inflammation (NC + H vs. I + HI). ^###^ *p* < 0.05, global effect of pulmonary inflammation (NC + H vs. I + HI).

We did not observe any effect of pulmonary inflammation or hypoxia in the SDH activity of Type I or IIa fibres in soleus (Figure 7C). In addition, we report no differences in the GPDH activity (Figure 7E) or the SDH/GPDH ratio (Figure S6A) in soleus Type I fibres. In contrast, pulmonary inflammation increased the GPDH activity in soleus Type IIa fibres (+15.9%, *p* < 0.001, *η* ^2^_*p*_ = 0.48, Figure 7E). Nevertheless, there was no significant change in the SDH/GPDH ratio in Type IIa fibres of soleus in the animals exposed to pulmonary inflammation (−32.9%, *p* = 0.051, *η* ^2^_*p*_ = 0.26, Figure S6A). Neither environmental condition altered the plantaris SDH activity regardless of the fibre type (Figure 7D). Conversely, pulmonary inflammation induced a significant decline in the GPDH activity in the Types I and IIa fibres of plantaris (Type I: −31.8%, *p* < 0.05, *η* ^2^_*p*_ = 0.33, Figure 7F; Type IIa: −39.5%, *p* < 0.01, *η* ^2^_*p*_ = 0.52, Figure 7F) but not in Type IIx or IIb (Figure 7F). Finally, pulmonary inflammation led to a significant increase in the SDH/GPDH ratio across all the fibre types of plantaris (Figure S6B).

## 4 Discussion

Consistent with our hypothesis, chronic hypoxia impaired predominantly plantaris leading to mitochondrial dysfunction, evidenced by a decline in CI‐supported respiration and HAD activity, accompanied by a reduction in muscle weight and the CSA of Type IIb fibres. Conversely, soleus exhibited greater resilience to hypoxia, preserving both its mass and respiratory properties, despite an elevated ROS emission. Chronic pulmonary inflammation triggered maladaptations in both muscles, as indicated by a dysregulation in mitochondrial calcium handling exclusively in soleus and divergent metabolic shifts in both soleus (a switch towards glycolysis in Type IIa fibres) and plantaris (a lower reliance on glycolysis in Type I and IIa fibres and a switch towards oxidative phosphorylation in Types IIx and IIb fibres). Moreover, we have demonstrated that these alterations in skeletal muscle mitochondrial function and metabolic regulation can occur independently of major fibre type shifts.

### 4.1 Effects of Chronic Hypoxia

At an FiO2 of 10%, the resulting hypoxia is generally considered moderate at the tissue level, particularly in skeletal muscle, where oxygenation remains above the threshold of severe hypoxia due to efficient perfusion and oxygen extraction. Nevertheless, hypoxia induced several changes in both muscles. In plantaris, the reduction in ADP‐stimulated CI‐driven respiration aligns with earlier studies demonstrating an impairment in mitochondrial respiratory capacity following chronic hypoxia [12, 21]. This may represent chronic adaptations to the reductive stress imposed by cellular hypoxia which leads to a buildup of reducing equivalents such as NADH and FADH_2_ and over‐reduction of the inner mitochondrial membrane‐embedded electron carriers [11]. This redox imbalance can increase electron leak especially at the level of CI and CIII [11, 22]. As chronic hypoxia did not elicit any increase in plantaris H_2_O_2_ emission, it can be suggested that these changes represent a coordinated downregulation of oxidative metabolism to mitigate oxidative damage. A potential mechanism underlying this adaptation may partially involve the induction of NADH dehydrogenase ubiquinone 1 alpha subcomplex 4‐like 2 (NDUFA4L2) by hypoxia‐inducible factor 1α (HIF‐1α) [23, 24, 25]. Although we did not measure NDUFA4L2 expression, its induction under hypoxia has been demonstrated to dampen oxidative phosphorylation (OXPHOS) and ROS production in a wide variety of tissues including skeletal muscle [25], neonatal cardiomyocytes and brain tissue [23] as well as in hypoxic domains of cancer cells [24, 26].

The hypoxia‐induced decrease in plantaris HAD activity could reflect a diminished utilisation of fatty acids for ATP production and is consistent with past studies [27, 28]. This may be the result of an overall downregulation of all the mitochondrial pathways to prevent further provision of NADH and FADH_2_. We can also hypothesise about an inhibition of pyruvate dehydrogenase due to induction of pyruvate dehydrogenase kinase 1 by chronic hypoxia [29] that would thereby lower Krebs cycle activity and subsequently lead to less demand for acetyl‐CoA from fatty acids. This lower mitochondrial activity does not appear to be compensated by a higher glycolytic flux as hypoxia did not upregulate GPDH or LDH activity (Figure S4) in plantaris. Therefore, the energy availability might have been limiting with consequences on ATP‐dependent processes such as protein synthesis. This is consistent with the reduction in plantaris weight and the CSA of Type IIb fibres under hypoxia. Additionally, although we observed a decrease in food intake during the first 3 weeks of the hypoxic exposure, chronic hypoxia has been previously reported to exert hypophagia‐independent muscle atrophy [13, 17].

In contrast with plantaris, mitochondrial respiratory capacity and mass were preserved in soleus confirming that oxidative muscles are more resilient to chronic hypoxia. These findings are in the same line with earlier data demonstrating unchanged OXPHOS in soleus after 14 days at 10% FiO_2_ [30]. The soleus' postural role may necessitate sustained mitochondrial function, which would also explain its mass preservation. However, the increase in H_2_O_2_ emission following GM administration appears to be a trade‐off for preserving OXPHOS. To determine whether this rise stems from an intrinsic alteration in ROS generation or a proportional increase in CI basal respiration, H_2_O_2_ emission rates were normalised with the corresponding State 2 CI‐mediated O_2_ consumption. Our results revealed a higher ratio in soleus under hypoxia, suggesting a greater fraction of electrons leaking to form ROS per O_2_. This observation may reflect a pathological ROS leak potentially explained by impaired H_2_O_2_ scavenging by catalase or glutathione peroxidase [31]. Conversely, the unaltered mPTP sensitivity and the maintenance of mitochondrial respiration and muscle mass in hypoxic soleus suggest that this higher ROS production was without damaging effects. Although we did not assess manganese superoxide dismutase 2 activity, we can speculate that its activity may have increased to convert excess O_2_•‐ to the less reactive H_2_O_2_, representing a protective adaptation to attenuate mPTP and mitochondrial DNA damage. Interestingly, the differences observed between the soleus and plantaris muscles may be due to their distinct fibre type composition. The plantaris is mainly composed of Type II fibres, which have a larger diameter and a lower capillary density compared to Type I fibres, potentially making it more sensitive to decreases in oxygen availability.

### 4.2 Effects of Chronic Pulmonary Inflammation

Inflammation induced a modest decrease in Δ body weight that was not significant when assessed on a weekly basis. These results suggest a progressive cumulative effect due to inflammation rather than an acute response occurring during the first week as seen with hypoxia. Interestingly, the absence of hypophagia in animals subjected to inflammation suggests that this weight loss may be attributed to increased energy expenditure, and therefore indicating a change in the metabolism. Although there was no effect of pulmonary inflammation on mitochondrial respiration of plantaris, we observed a downregulation in GPDH activity in Types I and IIa fibres without any changes in their SDH activity leading to a parallel higher SDH/GPDH ratio. In contrast, given that there were no differences in the GPDH activity of Types IIx and IIb fibres, their higher SDH/GPDH ratios suggest a shift towards oxidative metabolism. This is a novel finding implying that pulmonary inflammation may force the highly glycolytic fibres of plantaris to a metabolic state that is distant from its intrinsic functional capacity that may subsequently impact its peak power output. In addition, despite the increased reliance on oxidative metabolism, it is likely that the underdeveloped capillary network, lower levels of myoglobin and low levels of antioxidants defences observed in Types IIx and IIb fibres [32] may structurally constrain their capacity to support OXPHOS.

In soleus, we observed a decrease in mitochondrial CRC following pulmonary inflammation indicating faster mPTP opening. This effect was prevented after inhibition of CyP‐D by CsA, implying that this regulatory component of the mPTP may be directly modulated by pulmonary inflammation. Although we are the first to demonstrate increased mPTP sensitivity in skeletal muscle following pulmonary inflammation, comparable findings emanate from studies in muscle of COPD patients [6] or ageing muscles susceptible to chronic low‐grade inflammation [33]. The mPTP opening is known to be involved in proapoptotic factors release [34, 35], followed by activation of proteolytic pathways and atrophy [36]. Thus, the absence of atrophy and decline in mitochondrial respiration may suggest that pulmonary inflammation provokes recurring, transient, low conductance pore openings that increase the sensitivity to Ca^2+^and constitutes a protective mechanism to reset soleus mitochondrial function. This alteration in calcium handling by soleus mitochondria may result from disrupted cytosolic Ca^2+^ homeostasis and sarcoplasmic reticulum (SR) stress initiated by proinflammatory mediators. For instance, Qaisar et al. [37] reported higher SR stress coupled with sarcoplasmic reticulum Ca^2+^‐ATPase disruption in muscle of asthmatic patients. That promotes cytosolic Ca^2+^ accumulation and elevates mitochondrial Ca^2+^ uptake, leading to matrix Ca^2+^ overload, which sensitises the mPTP [38]. Whether these recurrent low conductance openings may eventually transition to the more deleterious high‐conductance mPTP openings [39] warrants further investigation.

Interestingly, we report that GPDH activity increased in Type IIa fibres of soleus following pulmonary inflammation suggesting a shift towards a more glycolytic profile that may have detrimental functional consequences such as earlier fatigue during posture maintenance. Our findings align with increased hexokinase 2 mRNA expression in muscle of COPD patients presenting elevated tumour necrosis factor α (TNF‐α) expression [40] and a glycolytic shift in myotubes exposed for 24 h to LPS [41] These alterations may stem from inflammation‐induced impairments in mitochondrial biogenesis [40, 42] and/or upregulation in mitophagic flux [42, 43] resulting in reduced mitochondrial content in Type IIa fibres and subsequent switch towards glycolysis.

### 4.3 Combined Effect of Chronic Hypoxia and Pulmonary Inflammation

The original combination of hypoxia with pulmonary inflammation enabled us to investigate whether the simultaneous presence of these two stressors exacerbates skeletal muscle dysfunction beyond the effects of each stimulus alone. Our statistical analysis did not reveal any significant interactions suggesting that the combination of hypoxia and pulmonary inflammation does not result in additive effects that would compound muscle dysfunction. In contrast, our findings underscore that the two stimuli operate through distinct pathways that potentially compete with each other to elicit a response that is muscle‐ and parameter‐specific. For instance, hypoxia appears to prevail over pulmonary inflammation with regards to plantaris CI‐mediated State 3 respiration and HAD activity, whereas pulmonary inflammation overrides hypoxia effects on soleus CRC. This likely reflects the opposing HIF‐1α vs. TNF‐α molecular signalling imposed by the two conditions and is consistent with previous work from our group showing distinct modulation of proteolysis and proteosynthesis pathways by hypoxia and pulmonary inflammation [16].

### 4.4 Strengths and Limitations

A major strength of our study was the simultaneous assessment of fibre typology and mitochondrial function in soleus and plantaris. Although skeletal muscle dysfunction in COPD has been proposed to stem from changes in fibre typology [19], we only report a rise in the percentage of Plantaris IIx fibres in response to pulmonary inflammation. Since no other differences were observed, it is challenging to predict whether this rise in Type IIx numbers represents a switch towards a more oxidative or glycolytic phenotype. Collectively, these results imply that mitochondrial intrinsic maladaptations may play a key role in the COPD‐associated muscle dysfunction. A key strength of this study was the evaluation of the independent and combined effects of inflammation and hypoxia. We deliberately chose this model to address several objectives. First, from a basic science standpoint, it enabled us to establish the unique fingerprint of each stressor, exploring whether the mitochondrial and metabolic responses are stress‐dependent. Second, the study of their combined effects allowed us to decipher whether their interactions are synergistic, antagonistic or independent providing a new insight of the complex pathophysiology seen in these patients.

A limitation of our study is that we have not disentangled the proportion of metabolic and mitochondrial changes attributed to hypophagia associated with hypoxia. One would suggest the inclusion of a pair‐fed NC group alongside the ad libitum group. However, due to our simultaneous investigation of pulmonary inflammation that does not induce changes in food intake and the fact that hypoxia is intrinsically associated with alterations in food intake, we opted to feed the NC group ad libitum and observe the global effects of hypoxia. Furthermore, although COPD is a disease mostly encountered in the ageing population [44], the use of young adult rats might have underestimated the magnitude of detrimental responses to hypoxia and pulmonary inflammation. One limitation of this study is the absence of a measure of spontaneous locomotor activity in the animals, which could contribute to explaining the effects observed in the study. Finally, only male rats were used and therefore our results are only applicable to male populations. Considering the sex differences in baseline mitochondrial bioenergetics [45] and antioxidant defences [46], future studies in female populations should confirm our findings.

## 5 Conclusion

Using our original model of chronic hypoxia and pulmonary inflammation exposures, we showed muscle‐specific mitochondrial and metabolic responses to those two key factors of COPD, with no overlap in their mechanism of action. Chronic hypoxia impairs predominantly the glycolytic‐leaning plantaris compared to oxidative soleus, whereas chronic pulmonary inflammation remodels the metabolic landscape of both muscles and increases the mPTP sensitivity in soleus. These alterations occur in the absence of fibre type shifts thereby suggesting that intrinsic mitochondrial defects may play a key role in the muscle dysfunction observed in COPD. Finally, distinct and opposing mechanisms of action between hypoxia and pulmonary inflammation highlight that the muscle response to these two stressors is not a simple sum of their isolated effects but rather a complex interaction where one factor may modify or obscure the other and thus targeted interventions for hypoxic versus inflammatory muscle defects should be developed.

## Funding

The authors have nothing to report.

## Conflicts of Interest

The authors declare no conflicts of interest.

## Supporting information


**Figure S1:** Respiratory control ratio in isolated mitochondria from soleus and plantaris muscle after exposure to hypoxia and/or inflammation. Soleus (A) and plantaris (B) RCR (state 3/state 4) with CI (GM), CII (S) and CI + CII (GMS) substrates. Circles indicate individual data points. Lines represent means and error bars indicate SD. RCR, respiratory control ratio.
**Figures S2:** CIV respiration in isolated mitochondria from soleus and plantaris muscle after exposure to hypoxia and/or inflammation. Soleus (A) and plantaris (B) uncoupled mitochondrial respiration measured by oxygraphy using AA, ascorbate, TMPD and DNP. Circles indicate individual data points. Lines represent means and error bars represent SD.
**Figures S3:** Soleus (A) and plantaris (B) mitochondria H_2_O_2_ release with GM or S were normalised to CI or CII basal (substrate only) oxygen consumption, respectively. Circles indicate individual data points with some of them obscured due to overlapping values. Lines represent means and error bars represent SD. Broken y‐axes were used to improve visualisation of values across different measurement ranges. ** *p* < 0.01, global effect of hypoxia (NC + I vs. H + HI).
**Figures S4:** Effect of hypoxia and/or inflammation exposure on plantaris LDH activity. LDH activity was measured in the cytosolic fraction of plantaris. Circles indicate individual data points. Lines represent means and error bars indicate SD.
**Figures S5:** Effect of hypoxia and/or inflammation exposure on soleus and plantaris hybrid fibre typology. Percentage of type I/IIa fibres in soleus (A) and type I/IIa, IIa/IIx and Iix/IIb fibres in plantaris (B). Circles indicate individual data points. Lines represent means and error bars indicate SD. Broken y‐axes were used to improve visualisation of values across different measurement ranges.
**Figures S6:** Effects of hypoxia and/or inflammation exposure on SDH/GPDH activity ratio of soleus and plantaris. SDH/GPDH ratio in type I and type IIa fibres of soleus (A) and type I, type IIa, IIx and type IIb fibres of plantaris (B). Circles indicate individual data points. Lines represent means and error bars indicate SD. Broken y‐axes were used in panel B to improve visualisation of values across different measurement ranges. **#:** *p* < 0.05, global effect of pulmonary inflammation (NC + H vs. I + HI).
