# Supplementary material for: Skeletal Muscle Fibre Type Determines Mitochondrial and Metabolic Responses to Hypoxia and Pulmonary Inflammation in Young Adult Rats
Source: J Cachexia Sarcopenia Muscle. 2026 Sep 23;17(5):e70382. doi: 10.1002/jcsm.70382 (PMC13601756; doi:10.1002/jcsm.70382)
Supplement: Supplementary file 1 [file JCSM-17-e70382-s001.docx]

**Control	Hypoxia	Inflammation	Hypoxia + Inflammation**

***Fig.S1. Respiratory control ratio in isolated mitochondria from soleus and plantaris muscle after exposure to hypoxia and/or inflammation****. Soleus (A) and plantaris (B) RCR (state 3/state 4) with CI (GM), CII (S) and CI + CII (GMS) substrates. Circles indicate individual data points. Lines represent means and error bars indicate SD. RCR, respiratory control ratio.*

**Control	Hypoxia	Inflammation	Hypoxia + Inflammation**

***Fig.S2. CIV respiration in isolated mitochondria from soleus and plantaris muscle after exposure to hypoxia and/or inflammation.*** *Soleus (****A****) and plantaris (****B****) uncoupled mitochondrial respiration measured by oxygraphy using AA, ascorbate, TMPD and DNP. Circles indicate individual data points. Lines represent means and error bars represent SD.*

**Control	Hypoxia	Inflammation	Hypoxia + Inflammation**

***Fig.S3. Soleus (A) and plantaris (B) mitochondria H_2_O_2_ release with GM or S were normalised to CI or CII basal (substrate only) oxygen consumption, respectively.*** *Circles indicate individual data points with some of them obscured due to overlapping values. Lines represent means and error bars represent SD.* *Broken y-axes were used to improve visualisation of values across different measurement ranges. ** p < 0.01, global effect of hypoxia (NC + I vs H + HI).*

**Control	Hypoxia	Inflammation	Hypoxia + Inflammation**

***Fig.S4. Effect of hypoxia and/or inflammation exposure on plantaris LDH activity.*** *LDH activity was measured in the cytosolic fraction of plantaris. Circles indicate individual data points. Lines represent means and error bars indicate SD.*

***Fig.S5. Effect of hypoxia and/or inflammation exposure on soleus and plantaris hybrid fibre typology.*** *Percentage of type I/IIa fibres in soleus (****A****) and type I/IIa, IIa/IIx and Iix/IIb fibres in plantaris (****B****). Circles indicate individual data points. Lines represent means and error bars indicate SD. Broken y-axes were used to improve visualization of values across different measurement ranges.*

***Fig.S6 Effects of hypoxia and/or inflammation exposure on SDH/GPDH activity ratio of soleus and plantaris.*** *SDH/GPDH ratio in type I and type IIa fibres of soleus (A) and type I, type IIa, IIx and type IIb fibres of plantaris (****B****). Circles indicate individual data points. Lines represent means and error bars indicate SD.* *Broken y-axes were used in panel B to improve visualization of values across different measurement ranges.* ***#:*** *p < 0.05, global effect of pulmonary inflammation (NC + H vs I + HI).*

**SUPPLEMENTARY DATA REFERENCE LIST**

41. Eggelbusch M, Shi A, Broeksma BC, Vázquez‐Cruz M, Soares MN, De Wit GMJ, et al. The NLRP3 inflammasome contributes to inflammation‐induced morphological and metabolic alterations in skeletal muscle. J Cachexia Sarcopenia Muscle. 2022 Dec;13(6):3048–61.
42. Remels AHV, Gosker HR, Schrauwen P, Hommelberg PPH, Sliwinski P, Polkey M, et al. TNF-α impairs regulation of muscle oxidative phenotype: implications for cachexia? FASEB J. 2010 Dec;24(12):5052–62.
43. Leermakers PA, Remels AHV, Langen RCJ, Schols AMWJ, Gosker HR. Pulmonary inflammation-induced alterations in key regulators of mitophagy and mitochondrial biogenesis in murine skeletal muscle. BMC Pulm Med. 2020 Dec;20(1):20.
44. Leermakers PA, Schols AMWJ, Kneppers AEM, Kelders MCJM, De Theije CC, Lainscak M, et al. Molecular signalling towards mitochondrial breakdown is enhanced in skeletal muscle of patients with chronic obstructive pulmonary disease (COPD). Sci Rep. 2018 Oct 9;8(1):15007.
45. Jarhyan P, Hutchinson A, Khaw D, Prabhakaran D, Mohan S. Prevalence of chronic obstructive pulmonary disease and chronic bronchitis in eight countries: a systematic review and meta-analysis. Bull World Health Organ. 2022 Mar 1;100(03):216–30.
46. Miotto PM, McGlory C, Holloway TM, Phillips SM, Holloway GP. Sex differences in mitochondrial respiratory function in human skeletal muscle. Am J Physiol-Regul Integr Comp Physiol. 2018 June 1;314(6):R909–15.
47. Tiberi J, Cesarini V, Stefanelli R, Canterini S, Fiorenza MT, La Rosa P. Sex differences in antioxidant defence and the regulation of redox homeostasis in physiology and pathology. Mech Ageing Dev. 2023 Apr;211:111802.
